# Dual-energy CT: minimal essentials for radiologists

**DOI:** 10.1007/s11604-021-01233-2

**Published:** 2022-01-04

**Authors:** Fuminari Tatsugami, Toru Higaki, Yuko Nakamura, Yukiko Honda, Kazuo Awai

**Affiliations:** Department of Diagnostic Radiology, Hiroshima University, 1-2-3 Kasumi, Minami-ku, Hiroshima, 734-8551 Japan

**Keywords:** Dual-energy CT, Computed tomography, Material decomposition, Detectability

## Abstract

Dual-energy CT, the object is scanned at two different energies, makes it possible to identify the characteristics of materials that cannot be evaluated on conventional single-energy CT images. This imaging method can be used to perform material decomposition based on differences in the material-attenuation coefficients at different energies. Dual-energy analyses can be classified as image data-based- and raw data-based analysis. The beam-hardening effect is lower with raw data-based analysis, resulting in more accurate dual-energy analysis. On virtual monochromatic images, the iodine contrast increases as the energy level decreases; this improves visualization of contrast-enhanced lesions. Also, the application of material decomposition, such as iodine- and edema images, increases the detectability of lesions due to diseases encountered in daily clinical practice. In this review, the minimal essentials of dual-energy CT scanning are presented and its usefulness in daily clinical practice is discussed.

## Introduction

Although the number of hospitals with dual-energy computed tomography (CT) scanners has increased, few facilities use the instruments in daily clinical practice. There are various analytical methods applicable to dual-energy CT, however, its clinical benefits are not widely applied. The dual-energy CT method scans the object at two different energies (tube voltages); it can be used to perform material decomposition based on the difference in the material-attenuation coefficients obtained at different energies. It also makes it possible to identify the characteristics of materials that cannot be evaluated on conventional single-energy CT scans. The ability to detect lesions encountered in clinical practice is improved by applying virtual monochromatic images or material decomposition, such as iodine- and edema images. Effective atomic number- and electron density analysis may reveal the properties of materials whose evaluation is difficult on conventional single-energy CT scans. Dual-energy CT scans may be useful in a wide range of specialties, e.g. emergency medicine, radiation therapy, and autopsy imaging. In this review, the basics of dual-energy CT and its usefulness in daily clinical practice are discussed.

## X-ray generation and energy spectrum

**Fig. 1:**
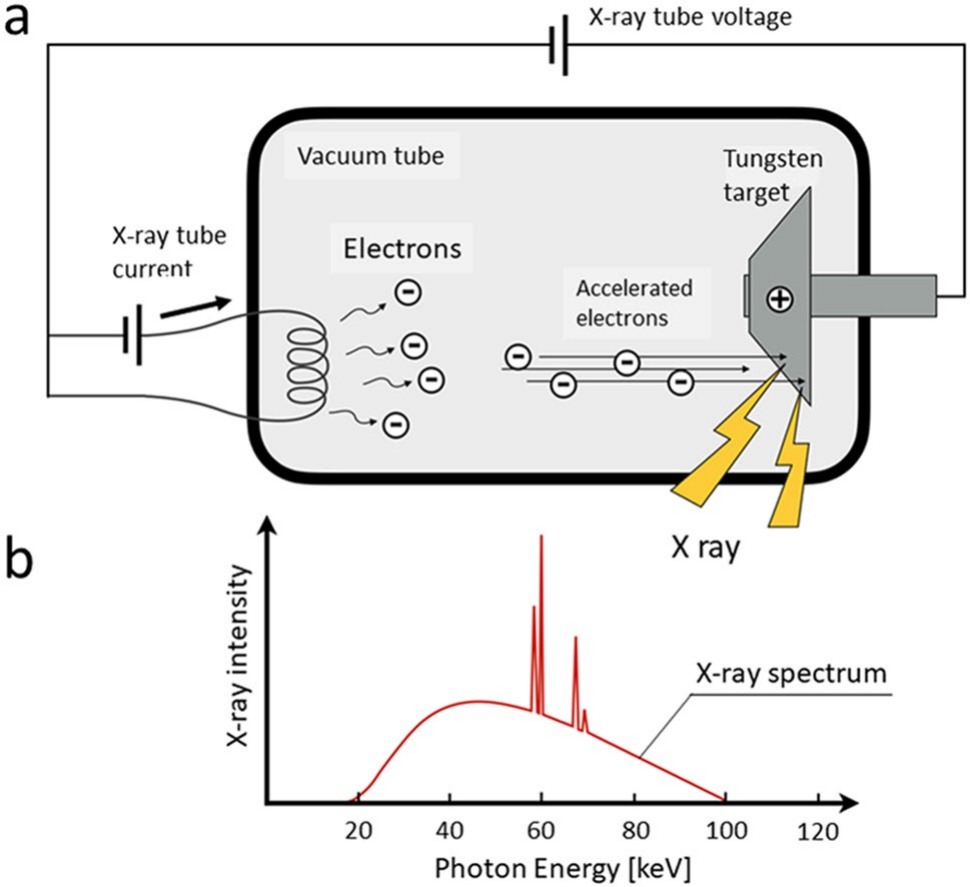
When accelerated electrons emitted from the cathode bombard the tungsten target anode, x-ray beams are produced (**a**). The x-ray beams are composed of photons in a broad continuum of energies that form the x-ray spectrum (**b**)

**Fig. 2:**
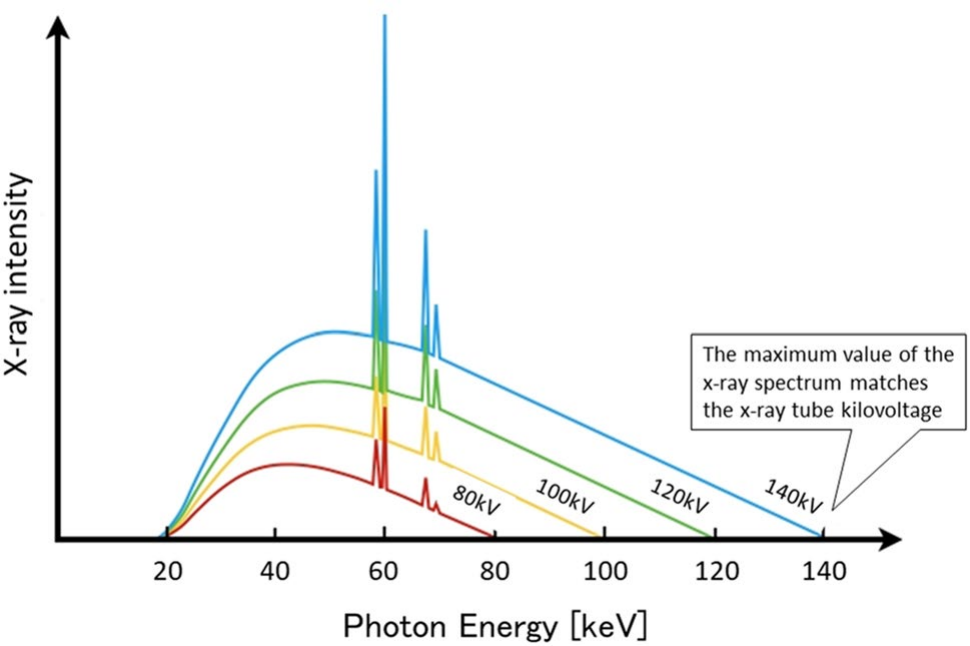
The x-ray spectrum varies depending on the tube voltage. The maximum value of the x-ray spectrum (keV) is equal to the x-ray tube kilovoltage (kV)

In CT scanners, the x-rays are generated in the x-ray tube (Fig. 1a). To produce the x-ray beams, an electron stream emitted from the cathode is focused into a narrow beam that bombards a small focal spot on the tungsten target anode [1]. The x-ray beams are composed of photons in a wide continuum of energies (kilo electron volt; keV); the beams are referred to as “polychromatic x-rays” that form the x-ray spectrum (Fig. 1b). The maximum value of the photon energy in the x-ray spectrum matches the x-ray tube kilovoltage (kV); if the tube voltage is 120 kV, the maximum energy of the spectrum is 120 keV (Fig. 2). The x-ray spectrum depends on the tube voltage; Fig. 2 shows x-ray spectra for x-ray tube voltages of 80, 100, 120, and 140 kV) [2].

The effective x-ray energy is often used as a representative value of a polychromatic x-ray photon spectrum; the effective energy is the energy of a polychromatic x-ray expressed as the energy of a monochromatic x-ray with equivalent interactions. Specifically, the effective energy is measured using an absorber composed of aluminum (Al) or copper (Cu). The CT attenuation number expressed as Hounsfield units (HU) at approximately 65–70 keV on virtual monochromatic images (VMIs) is equivalent to the HU on single-energy CT images acquired at 120 kV [3]. Therefore, VMIs acquired at 65–70 keV are almost equivalent to single-energy CT images obtained at 120 kV.

## Scanning and analysis methods

### Principles of dual-energy CT

**Fig. 3:**
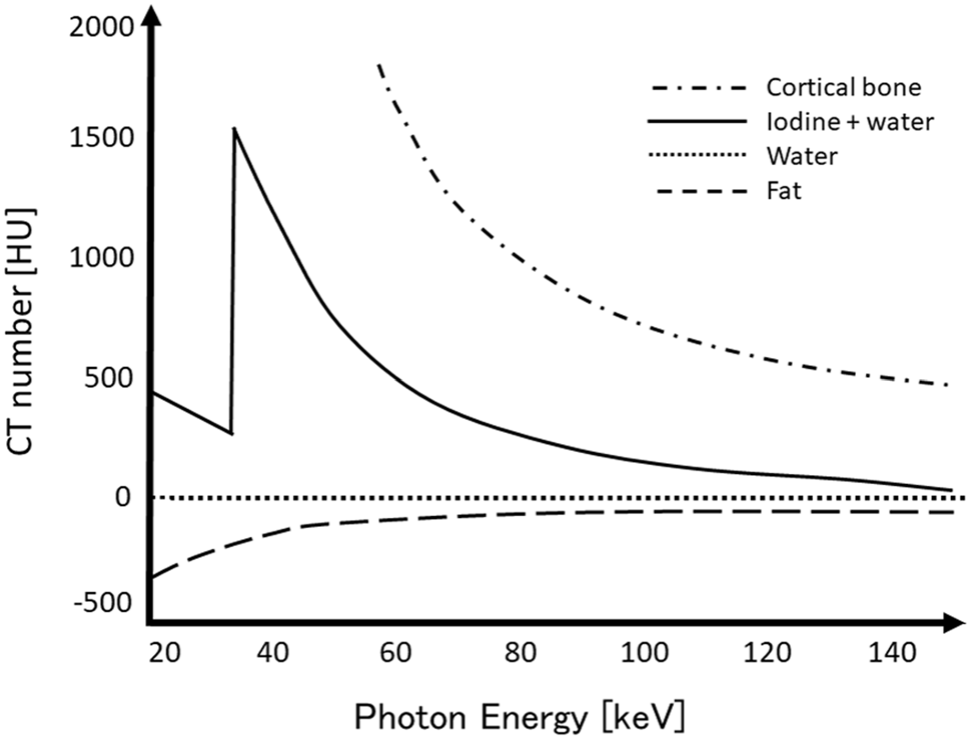
A material has different CT numbers at different energy levels. The degree of the difference depends on the material’s elemental composition

In general, a material has a different CT number at different energy levels [4–6]; the degree of this difference depends on the material’s elemental composition (Fig. 3). The CT number of a material relates to its linear attenuation coefficient [7] and is not unique for any given material. Materials can have a similar CT number even when their elemental composition is different.

**Fig. 4:**
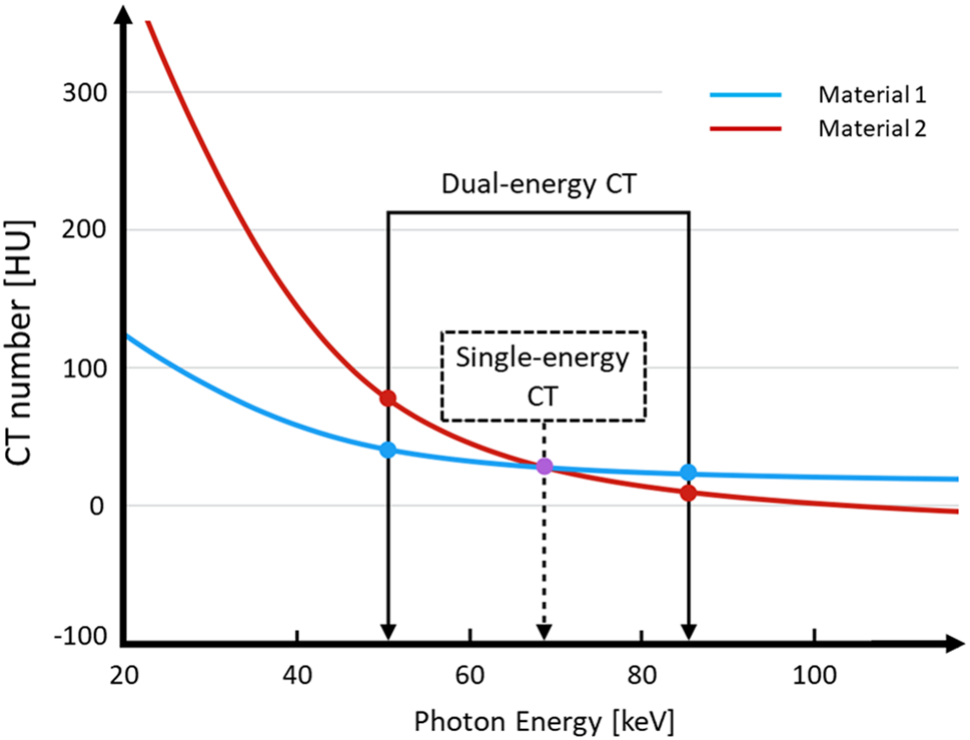
On conventional single-energy CT images, two materials can often not be distinguished due to considerable overlap in their CT numbers. On dual-energy CT scans, materials with different elemental compositions can be differentiated and quantified by comparing their CT numbers at two different energy levels

On conventional single-energy CT images, it is often difficult to distinguish between two materials (e.g. calcium and iodine) because there is a considerable overlap in their CT numbers [7]. Consequently, single-energy CT yields limited information on the material composition of tissues (Fig. 4). On dual-energy CT images, materials with different elemental compositions can be differentiated and quantified by comparing their CT number at the two different energy levels (Fig. 4).

### Types of dual-energy CT scanners

Vendors have produced scanners for clinical use that apply different dual-energy technologies [2, 8]. Two independent x-rays are used at the fast tube-voltage switching, sequential scan, and dual-source CT system. Commonly, 70–100 kVp and 135–150 kVp are routinely set for dual-energy CT scanning. Some vendors use only one x-ray source; the beam is separated into low- and high-energy spectra at the level of the detector (dual-layer system) or at the tube output (split filter system).

### Common requirements for dual-energy CT scanning

For accurate dual-energy analysis, images acquired with two different energies (voltages) should be temporally and spatially matched. The following are common requirements for dual-energy CT scanning [9].

(1) High- and low-energy data should be acquired simultaneously or with a small interval. A prolonged time gap results in a spatial mismatch between the two data sets due to patient movement, gastrointestinal peristalsis, or the flow of contrast material. (2) The energy difference between two data should be large. As dual-energy CT analysis is based on the contrast between the x-ray absorption of the two-energy data, a smaller energy difference results in a lower contrast-to-noise ratio. (3) The image quality, especially the image-noise level on low- and high-tube voltage scans, should be almost the same. If the image quality of one scan is poor, the quality of the final image will also be poor. It is desirable to increase the tube current for low-voltage scans. The better these requirements are satisfied, the better is the accuracy of dual-energy analysis.

### Dual-energy CT analysis methods

**Fig. 5:**
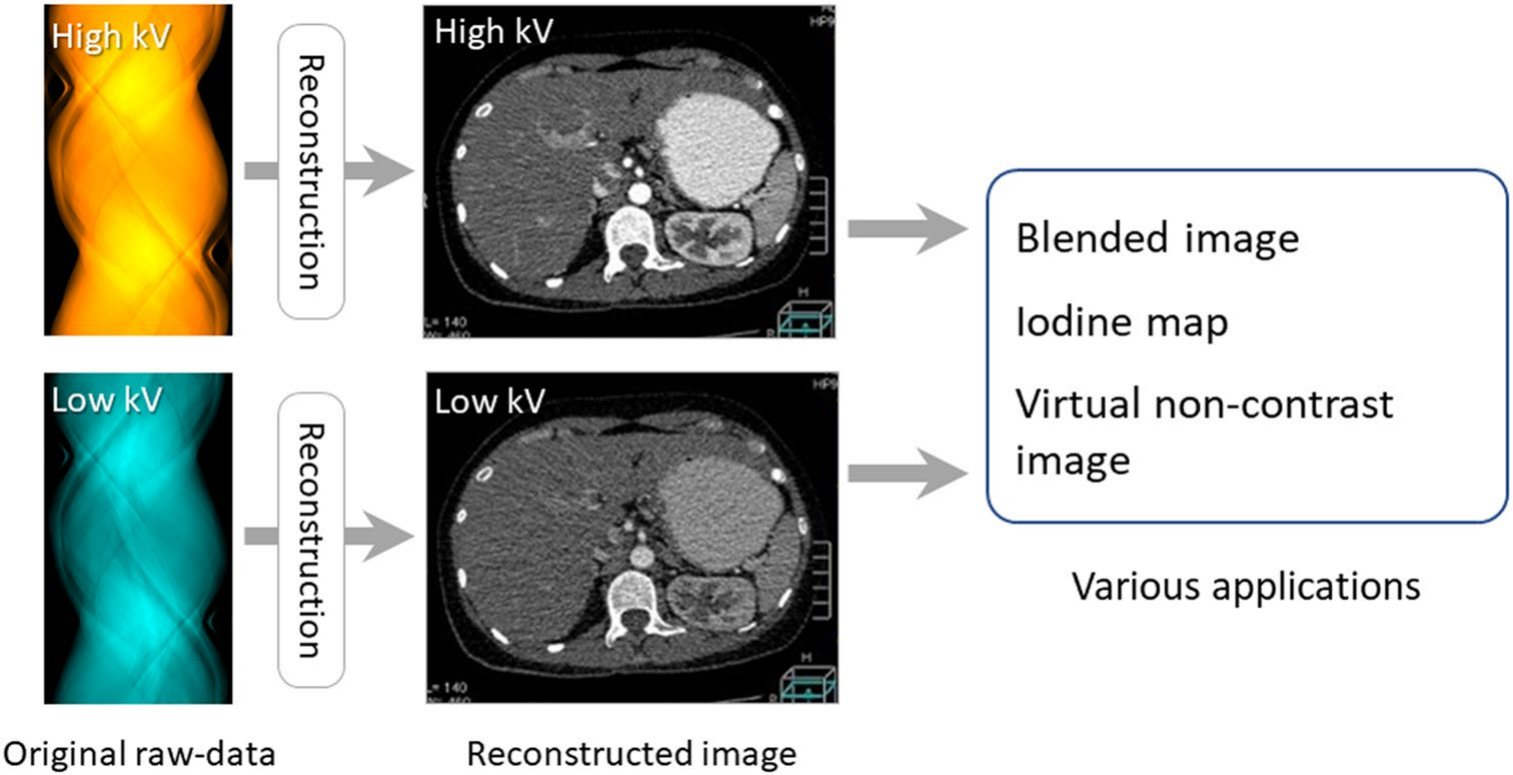
Image-based approach for dual-energy CT analysis. The x-ray paths at high- and low-tube voltages do not need to be perfectly matched. Dual-energy data are processed after the reconstruction of high- and low-energy images, then various applications are created. Dual-energy CT images created by image-based analysis contain various artifacts, e.g. beam hardening-, motion-, and helical artifacts

**Fig. 6:**
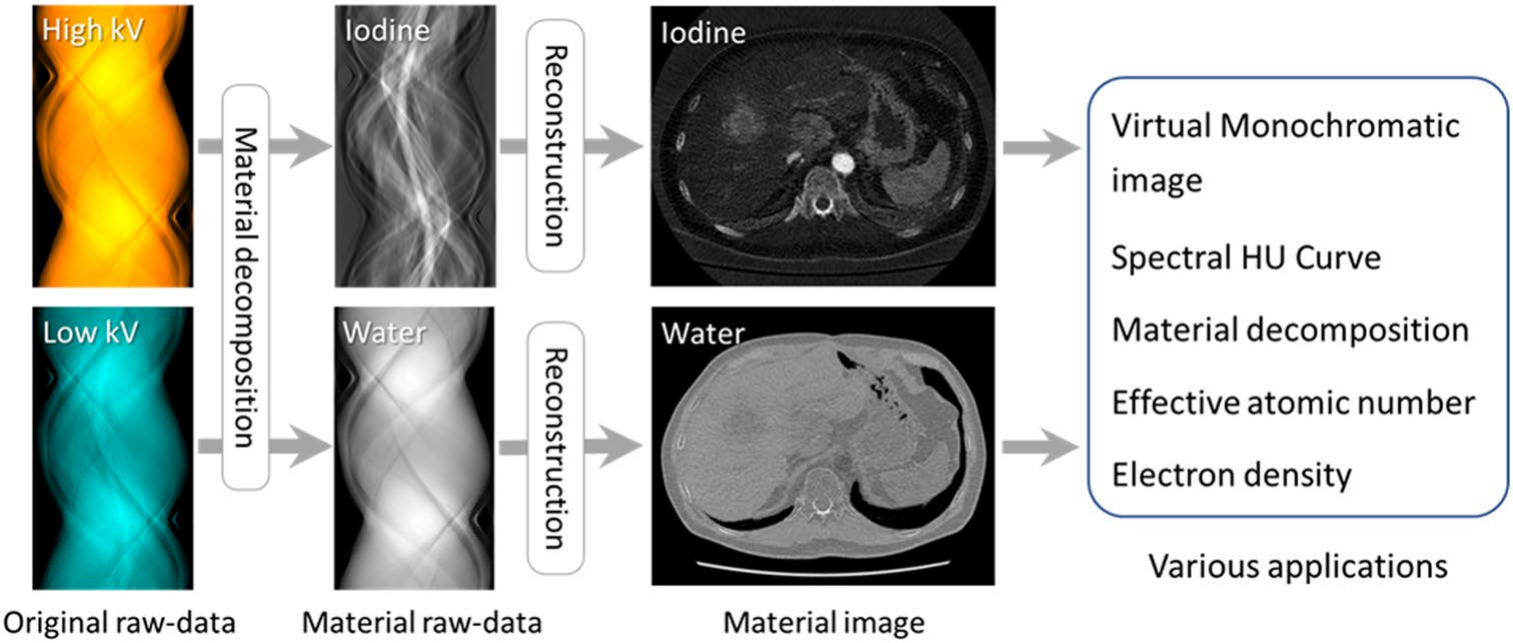
Raw data-based approach for dual-energy CT analysis. The x-ray paths at the high- and low-tube voltages must match exactly. Material raw data are processed directly by material decomposition, then image reconstruction is performed. The obtained CT applications have fewer beam-hardening effects and artifacts related to the CT reconstruction kernel than image-based analysis

Dual-energy analysis methods can be classified into image data-based analysis (Fig. 5) and raw data-based analysis (Fig. 6) [10]. Dual-energy scans are post-processed before (raw data-based analysis) or after (image-based analysis) the reconstruction of high- and low-energy images to create various dual-energy CT applications.

For image-based analysis (Fig. 5), the x-ray paths for the high- and low-tube voltages need not be perfectly matched as long as the two reconstructed images are spatially matched. Dual-energy data are processed after the reconstruction of the high- and low-energy images to create various dual-energy CT applications [11, 12]. The weighted average images at various tube voltages can be obtained by blending high- and low-energy images (blended image). Iodine-map images can be created by extracting the iodine (material decomposition); virtual non-contrast images by subtracting the iodine map images from the weighted average images. Dual-energy CT images created by image-based analysis contain various artifacts, e.g. beam hardening-, motion-, and helical artifacts. Consequently, they are less accurate than scans acquired with the raw-data based approach.

For raw data-based analysis (Fig. 6), the x-ray paths for the high- and low-tube voltages must match exactly. After material raw-data (iodine and water, or bone and water are the reference materials) are processed directly by material decomposition, image reconstruction is performed [11, 12]. The human body is considered to contain a mixture of two different materials, generally iodine and water, and the content of each material is calculated from the original raw-data set. Raw data-based analysis has a greater variety of dual-energy CT applications than image-based analysis. VMI-, electron density-, and effective atomic-number analyses require raw data analysis [13].

The choice between raw data- and image-based analysis depends on the dual-energy CT hardware. Currently, raw data-based analysis is used with fast tube-voltage switching-, sequential scanning-, and dual-layer detector systems. Dual-source CT scanners are used for image-based analysis [10].

### Advantages of raw data- over image-based analysis

Raw data-based analysis elicits lower beam-hardening effects and fewer artifacts related to the CT reconstruction kernel [14–16]. This results in more accurate CT number measurements in the scanned object.

**Fig. 7:**
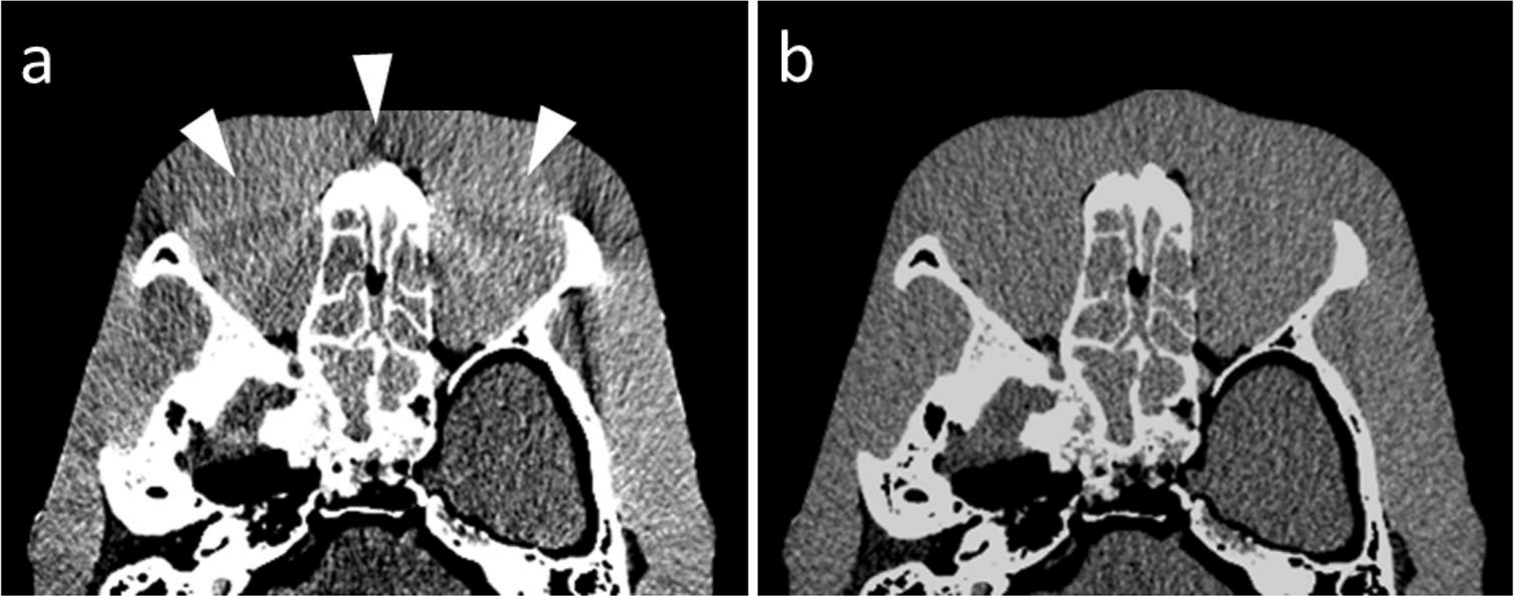
CT image processed with image-based analysis (**a**) and raw data-based analysis (**b**). In image-based analysis, beam-hardening artifacts from facial bones degrade the image quality (arrowheads). As the CT image processed with raw data-based analysis rather than image-based analysis exhibits lower beam-hardening artifacts, the acquired CT number would be accurate

Beam hardening on CT scans is attributable to the preferential attenuation of low- rather than high-energy x-ray photons as a polychromatic x-ray passes through the object. This can result in streaks and dark bands, particularly after passing through highly attenuated areas such as sites of severe calcification, sites with high concentrations of contrast material, and metallic objects such as stents and coils [17]. In raw data-based analysis, beam hardening is corrected during the generation of material projection data from the original projection data. Therefore, dual-energy CT images are less affected by beam-hardening artifacts (Fig. 7), and their analysis is more accurate than image-based analysis [18].

On the other hand, artifacts related to the CT reconstruction kernel such as blaring and over- and undershooting appear after image reconstruction. In raw data-based analysis, as dual-energy data are processed before image reconstruction, various dual-energy CT applications are less affected by these artifacts. Table 1 compares image- and raw data-based analyses.

**Table 1:** Comparison of image- and raw data-based analysis

	Analysis of dual-energy CT	
	Image-based analysis	Raw data-based analysis
Scanning	Projection-data at two energies that do not need to match	Projection-data at two energies must match
Postprocessing of dual-energy data	After reconstruction	Before reconstruction
Dual-energy CT applications	Limited applications	Wide variety of applications
Image quality	Contains various artifacts	Less affected by various artifacts

## Single energy-like images

### Virtual monochromatic images

Polychromatic x-ray beams delivered with single-energy CT are composed of photons at many energy levels that form the x-ray spectrum. VMIs are images that simulate CT images obtained with monochromatic x-rays of arbitrary energy.

**Fig. 8:**
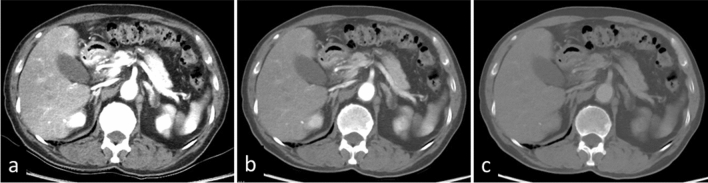
Virtual monochromatic images obtained at 40 (**a**), 70 (**b**), and 140 keV (**c**) (window level/width; 30/580 HU). On dual-energy CT scans, a monochromatic image, looking as if it had been acquired with single energy (keV), can be synthesized arbitrarily. The CT attenuation number on approximately 65–70 keV virtual monochromatic images is equivalent to single-energy CT scans acquired at 120 kV. The iodine contrast increases as the energy level decreases

In dual-energy processing, the linear attenuation coefficient (μ) within a certain voxel can be expressed by the formula

$$\mu \left( E \right) = \mu_{1} \left( E \right)c_{1} + \mu_{2} \left( E \right)c_{2} ,$$

where the mass density of the two basis materials (*c*_1_, *c*_2_) are estimated from material decomposition, and the linear attenuation coefficients of the two basis materials [μ_1_(*E*), μ_2_(*E*)] are known. The CT number at a certain energy level (keV) is defined by the formula

$${\text{CT number}}\left( E \right) = 1000\left[ {\mu \left( E \right) - \mu_{{{\text{water}}}} \left( E \right)} \right]/\mu_{{{\text{water}}}} \left( E \right),$$

where *μ*_water_ (*E*) is the linear attenuation coefficient of water. Using the two formulae, the CT number at arbitrary energy levels (keV) can be obtained (Fig. 8).

The CT attenuation number at approximately 65–70 keV on VMIs is equivalent to the number on single-energy CT scans acquired at 120 kV [3]. Therefore, VMIs in this energy range are often selected as the standard images. Generally, the image noise on VMIs obtained in this energy range is the lowest [3, 14].

**Fig. 9:**
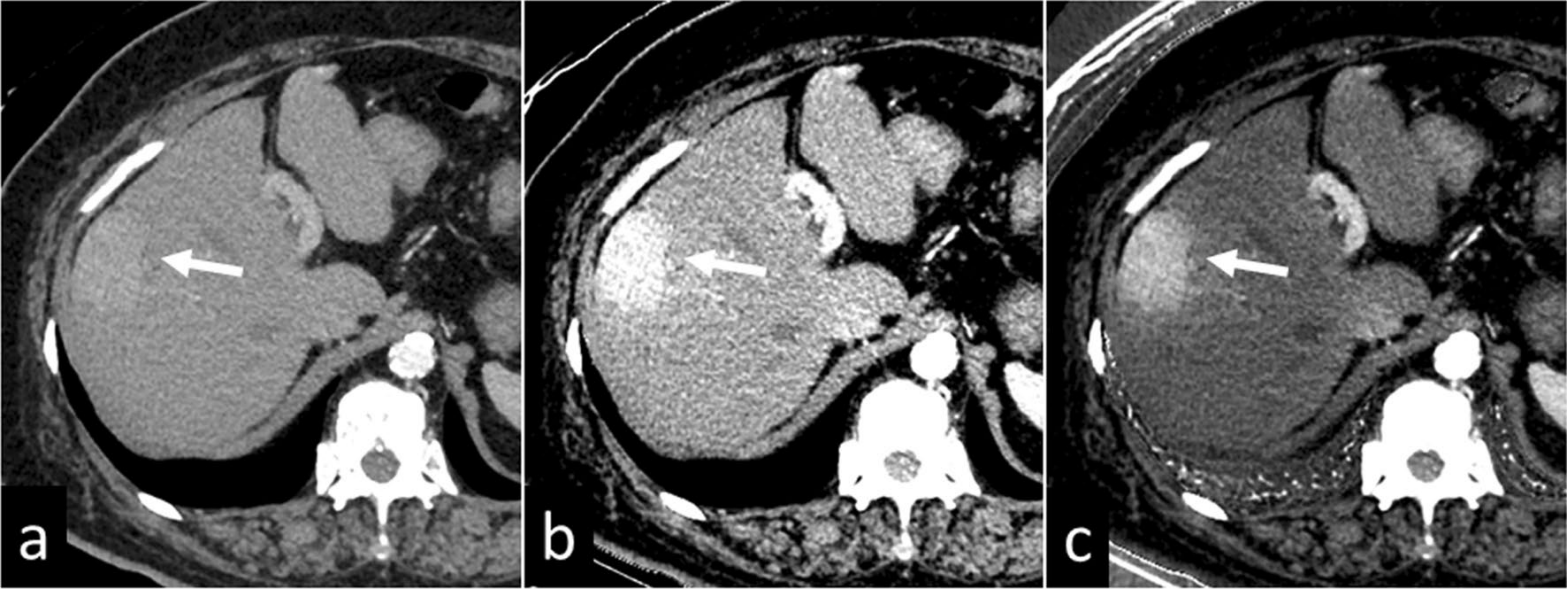
A 64-year-old woman with hepatocellular carcinoma. CT images during the arterial phase were obtained with a low contrast material dose (220 mgI/kg) due to renal insufficiency (eGFR, 21 ml min^–1^ 1.73 m^–2^). Visualization of the liver lesion is insufficient on the virtual monochromatic 70 keV image (**a**), whereas it is clearly detected on the monochromatic 40 keV image (**b**), and the iodine map (**c**)

As with single-energy CT scans performed at low-tube voltage (e.g. 80 or 100 kVp), the iodine contrast increases as the energy level of the VMI decreases (i.e. energy levels lower than 60 keV); this improves visualization of contrast-enhanced lesions. By taking advantage of this characteristic, VMIs at 40–50 keV generated from dual-energy CT scans allow for a contrast material dose reduction of 40–60% [19–21], this is especially important in patients with renal insufficiency (Fig. 9). As the image noise is increased on VMIs at lower keV settings, the application of a noise reduction technique, e.g. iterative reconstruction, is recommended.

**Fig. 10:**
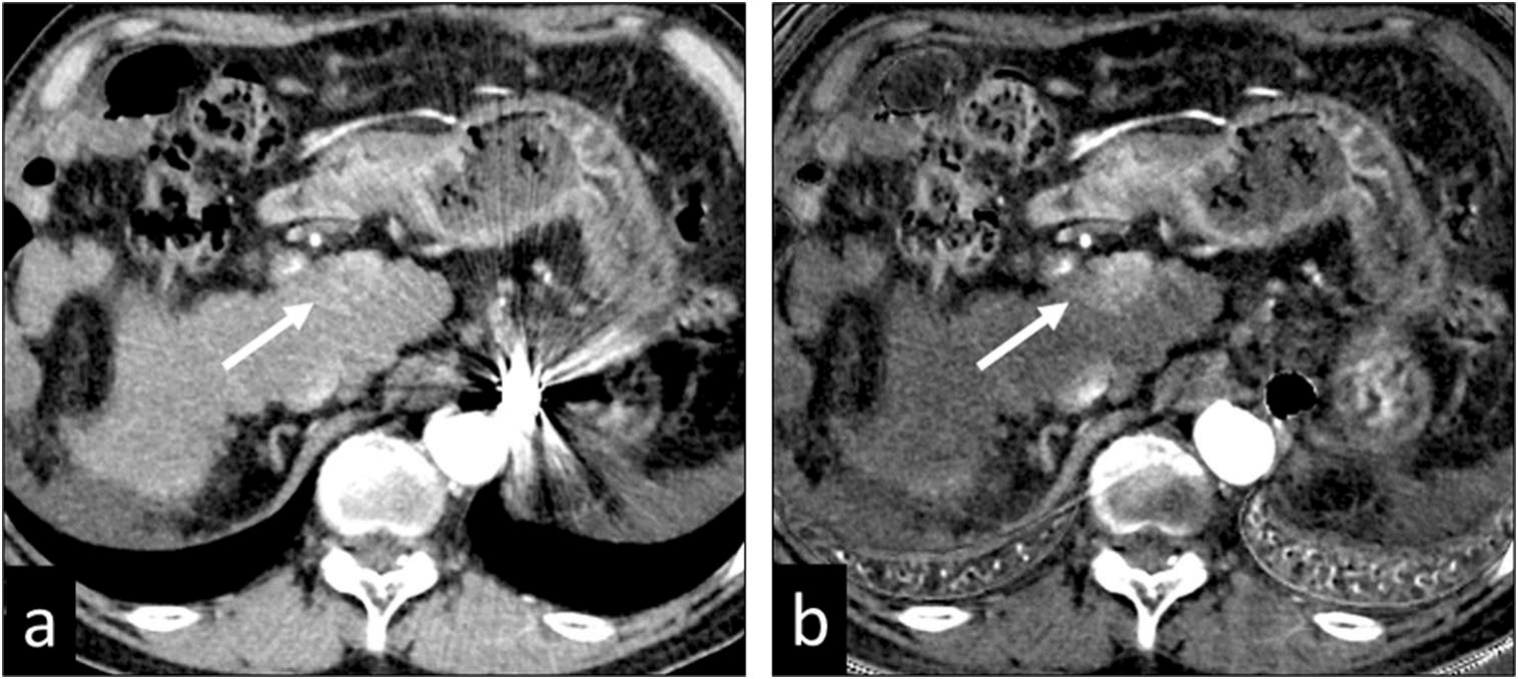
A 66-year-old man with hepatocellular carcinoma in the caudate lobe (arrows). On the 70 keV virtual monochromatic image (**a**), metal artifacts from the metallic coil implanted in the left inferior phrenic vein affect tumor detection. On the iodine map applied with metal artifact reduction software (**b**), the metal artifacts are reduced and the visibility of the tumor is considerably improved

When the energy level of VMIs increases (i.e., higher than 80 keV), the contrast between tissues is reduced, rendering metallic artifacts less noticeable. Nonetheless, to overcome severe artifacts from dense materials such as metallic clips, coils, and stents, we suggest the use of metal artifact reduction software (Fig. 10).

### Spectral HU curves

**Fig. 11:**
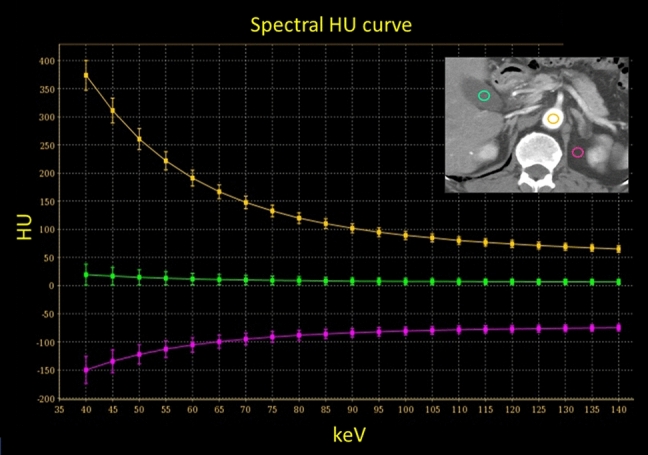
Spectral HU curves are obtained by setting a region of interest in tissue and plotting the average CT number at each monochromatic energy. The attenuation of high atomic number materials, such as iodine (insert, yellow circle) increases at lower energies, that of water is zero at all energies (insert, green circle), and that of fat decreases at lower energies (insert, red circle)

VMIs can be used to create spectral HU curves on a workstation. By setting a region of interest (ROI) in a tissue and plotting the average CT number in the ROI at each monochromatic energy (e.g. from 40 to 140 keV) of the VMI, spectral HU curves are obtained (Fig. 11). Since the shape of the curve varies with the mean attenuation characteristics in the ROI tissue, this facilitates the characterization of specific tissue types and is useful for component analysis and the acquisition of a differential diagnosis.

**Fig. 12:**
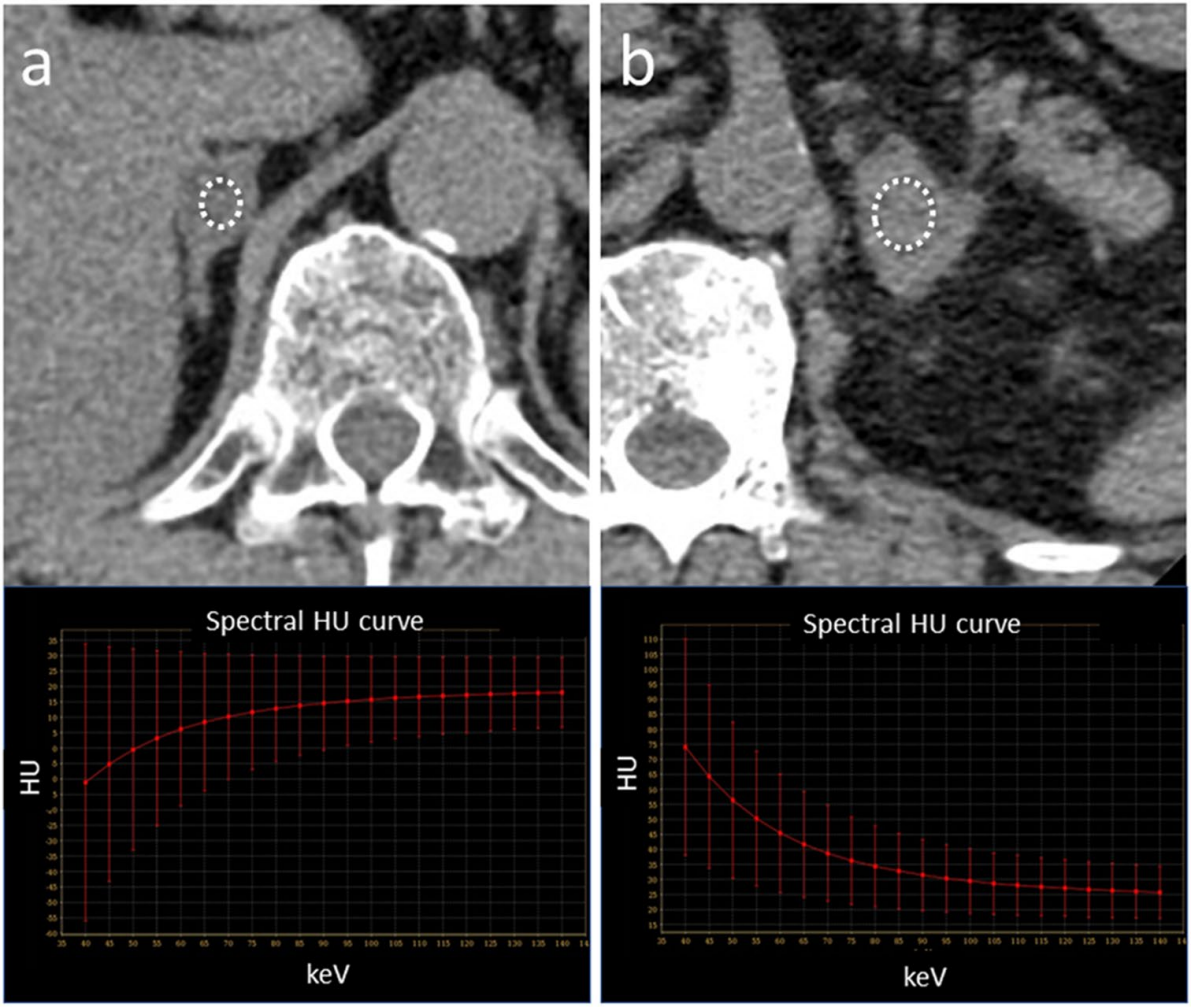
Axial monochromatic 70 keV images showing an adrenal adenoma (**a**) and an adrenal metastasis (**b**). Based on its CT number (HU = 19), the adenoma is not lipid-rich. At lower energy levels, the CT attenuation of the tumor decreases (**a**), suggesting that it contains fat. On the other hand, attenuation of the adrenal metastasis is increased at lower energy levels (**b**)

The attenuation of soft tissue and of high atomic number materials such as iodine and bone are increased at lower energies. The attenuation of water is zero at all energies; that of fat is decreased at lower energies (Fig. 11). The presence of fat is suggested when the curve pattern in the ROI of a specific tissue indicates decreased attenuation at lower keV. This observation helps in the diagnosis of fat-containing diseases, e.g. lipid-rich plaques, adrenal adenomas (Fig. 12), and angiomyolipomas.

## Material decomposition

Material decomposition images yield qualitative and quantitative information about the tissue composition. Two-, three-, and multi-material decomposition algorithms that can be applied to dual-energy CT are commercially available. We present material decomposition images commonly used in clinical practice, i.e. iodine-, virtual non-contrast-enhanced-, and edema images, and the liver fat volume fraction.

### Iodine images

Using three-material decomposition, iodine images, i.e. iodine-enhanced images generated by subtracting water from contrast-enhanced dual-energy CT images, are prepared. Iodine images, most commonly used to distinguish between enhanced and non-enhanced lesions, improve visualization of hyper- and hypo-vascular masses.

**Fig. 13:**
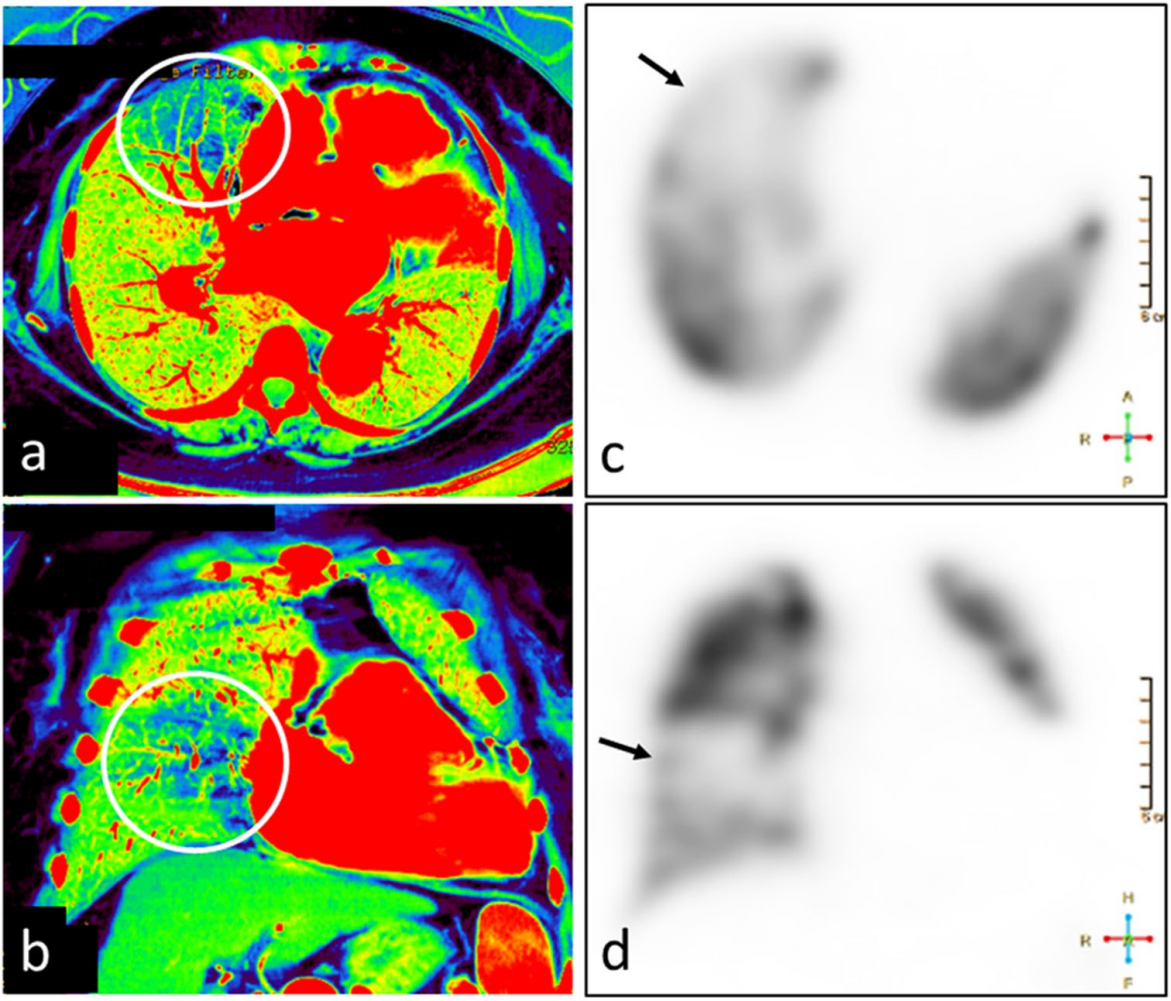
A 67-year-old woman with pulmonary-tumor thrombotic microangiopathy. The tumor embolism was too small for its detection on the contrast-enhanced CT scan. Catheterization demonstrated tumor embolism. Iodine maps (**a**, **b**) show areas with decreased blood flow in the right lung (circle), a finding consistent with a defect on lung perfusion scintigraphy (**c**, **d**) (arrows)

**Fig. 14:**
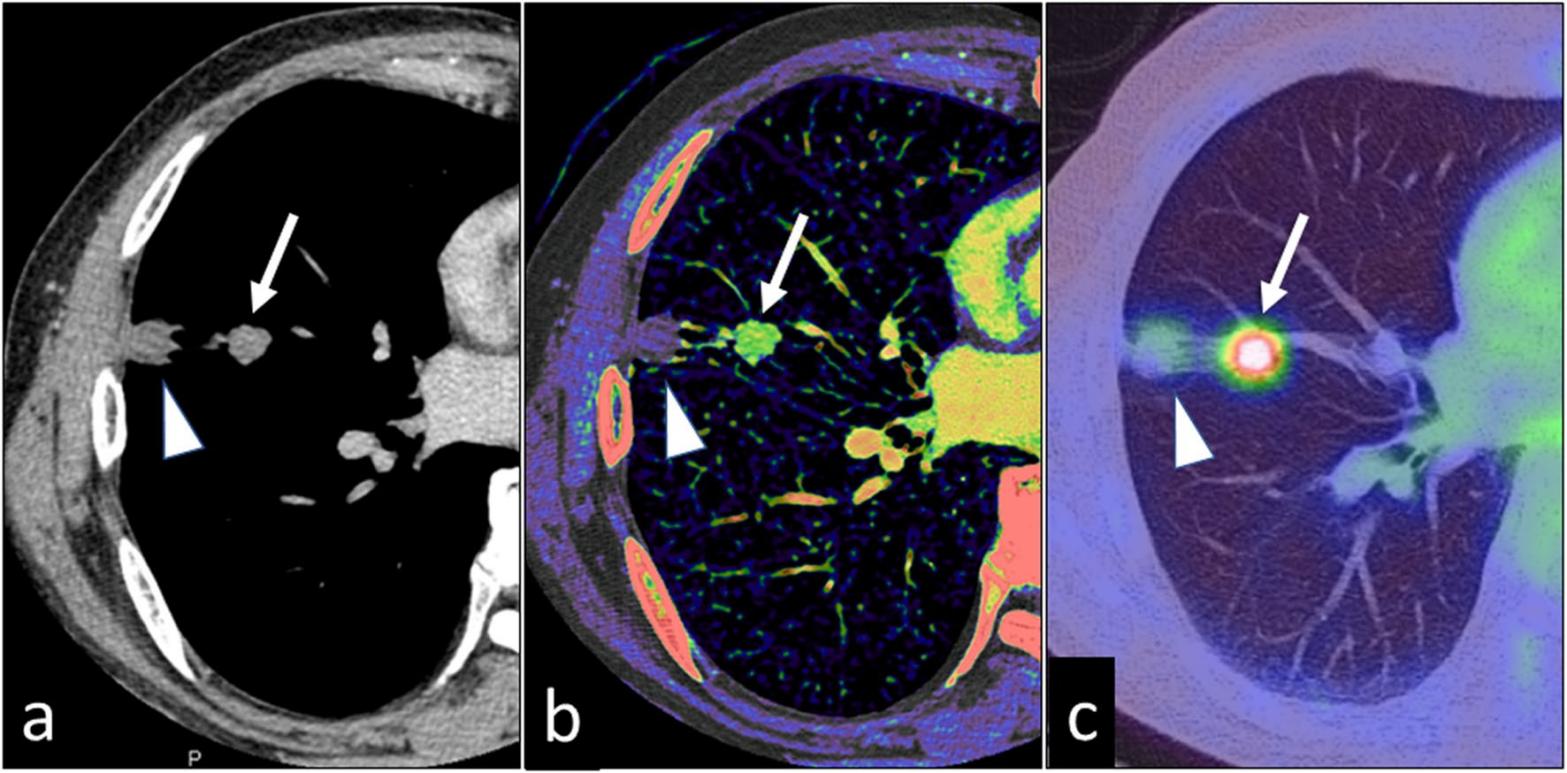
A 76-year-old man with two pulmonary nodules in the right lung. On the 70 keV virtual monochromatic image (**a**), the degree of enhancement is similar for both nodules. The iodine map (**b**) shows that the nodule at the proximal site (arrow) is highly vascular; the peripheral nodule (arrowhead) is not enhanced. Pathologically, the proximal nodule was identified as an adenocarcinoma and the peripheral nodule as an infarction. **c** PET-CT image (the maximum standardized uptake value of the tumor was 6.4)

The three-material decomposition algorithm enables the generation of a pulmonary blood volume (PBV) map that represents the iodine distribution in the lung parenchyma; it can be used as an indicator of pulmonary perfusion [22, 23]. PBV maps and iodine images help to identify pulmonary embolism-associated perfusion defects (Fig. 13). Also, as iodine images indicate the vascularity of pulmonary nodules, they contribute to their characterization (Fig. 14) [24].

The superior lesion-to-parenchyma contrast on iodine images improves lesion conspicuity and the delineation of lesion margins, thereby contributing to the reliable recognition of small lesions or only slightly attenuating tumors. The images also help to differentiate among enhanced-, non-enhanced-, and pseudo-enhanced tissue. Iodine-water material decomposition on dual-energy CT images facilitates estimation of the iodine concentration (mg/ml) in tissues [11].

**Fig. 15:**
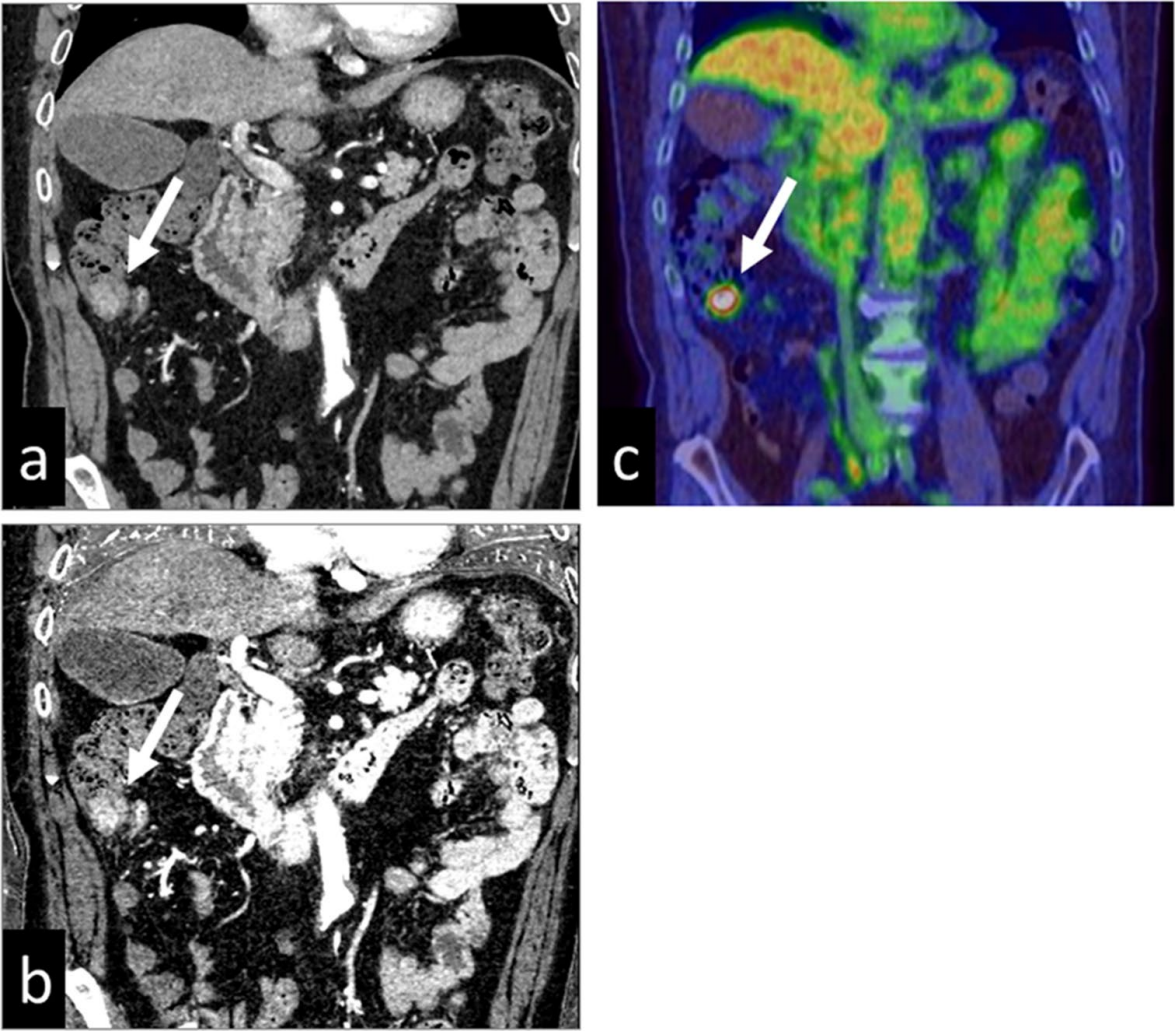
A 71-year-old man with cancer of the ascending colon. Virtual monochromatic image at 70 keV (**a**) and iodine map (**b**) during the arterial phase are shown. The iodine map yields better conspicuity than the monochromatic 70 keV image. **c** PET-CT image (the maximum standardized uptake value of the tumor was 6.1)

The detectability of gastric and colorectal tumors is improved on iodine images (Fig. 15), as is the differentiation between malignant and benign lesions [25, 26]. Iodine images are also useful in patients with acute abdomen such as small-bowel ischemia or gastrointestinal bleeding. They increase the conspicuity of hypo-attenuating segments in the bowel wall, thereby potentially improving the early detection of ischemia [27]. They can also help to identify subtle areas of contrast-medium extravasation for the accurate localization of the source of gastrointestinal bleeding [28].

**Fig. 16:**
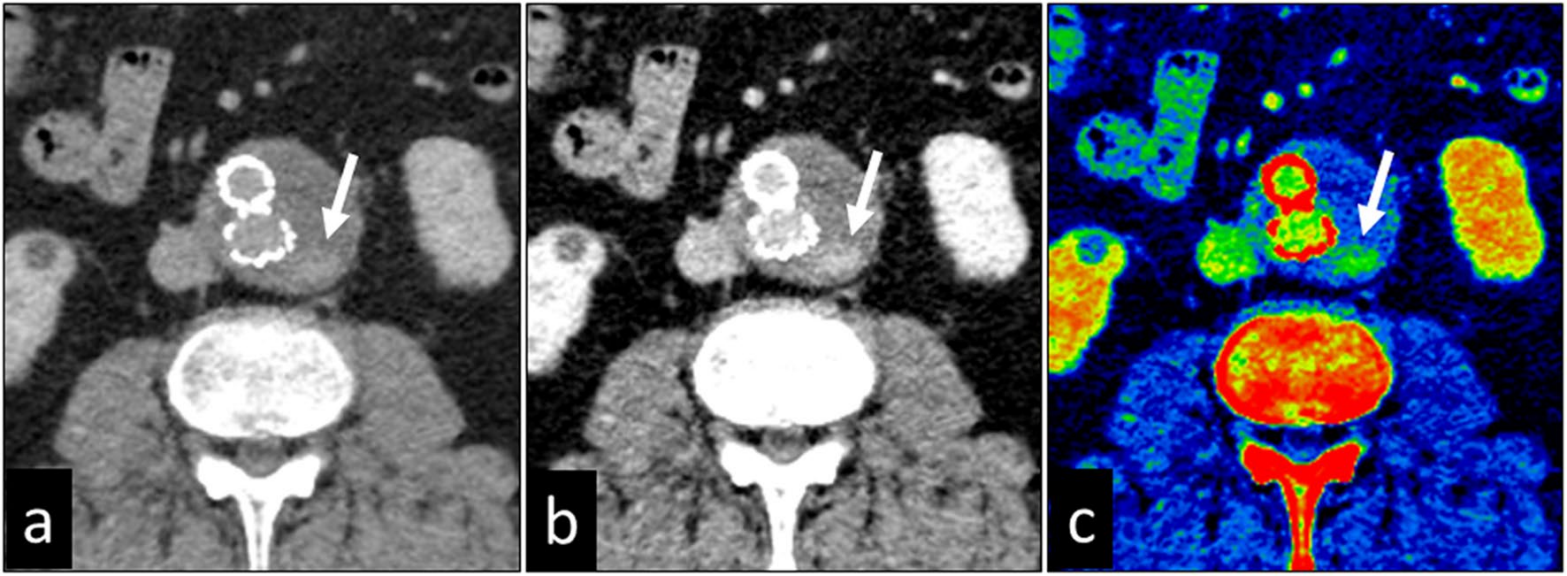
A 75-year-old man with endoleak after endovascular aortic repair. Virtual monochromatic images at 70 keV (**a**), 40 keV (**b**), and a color image of the iodine map (**c**) obtained with CT angiography are shown. The vessel contrast and the endoleak delineation (arrow) are better on the 40 keV image and the iodine map than on the monochromatic 70 keV image

Contrast-enhanced dual-energy CT scans are valuable for the detection and denial of endoleaks after endovascular aortic repair (EVAR) [29, 30]. While VMIs obtained at lower energy increase the vessel contrast, blooming- and metallic artifacts decrease the image quality. Iodine images, on the other hand, improve endoleak conspicuity without an increase in blooming artifacts (Fig. 16).

### Virtual non-contrast enhanced image

Using three material decomposition, virtual non-contrast-enhanced (VNC) images can be generated by subtracting the iodine component from the contrast-enhanced dual-energy CT image. Such VNC images facilitate the differentiation of calcifications or high-attenuation materials from iodine-enhanced tissues. The acquisition of VNC images may obviate radiation exposure when unenhanced CT scans are needed.

**Fig. 17:**
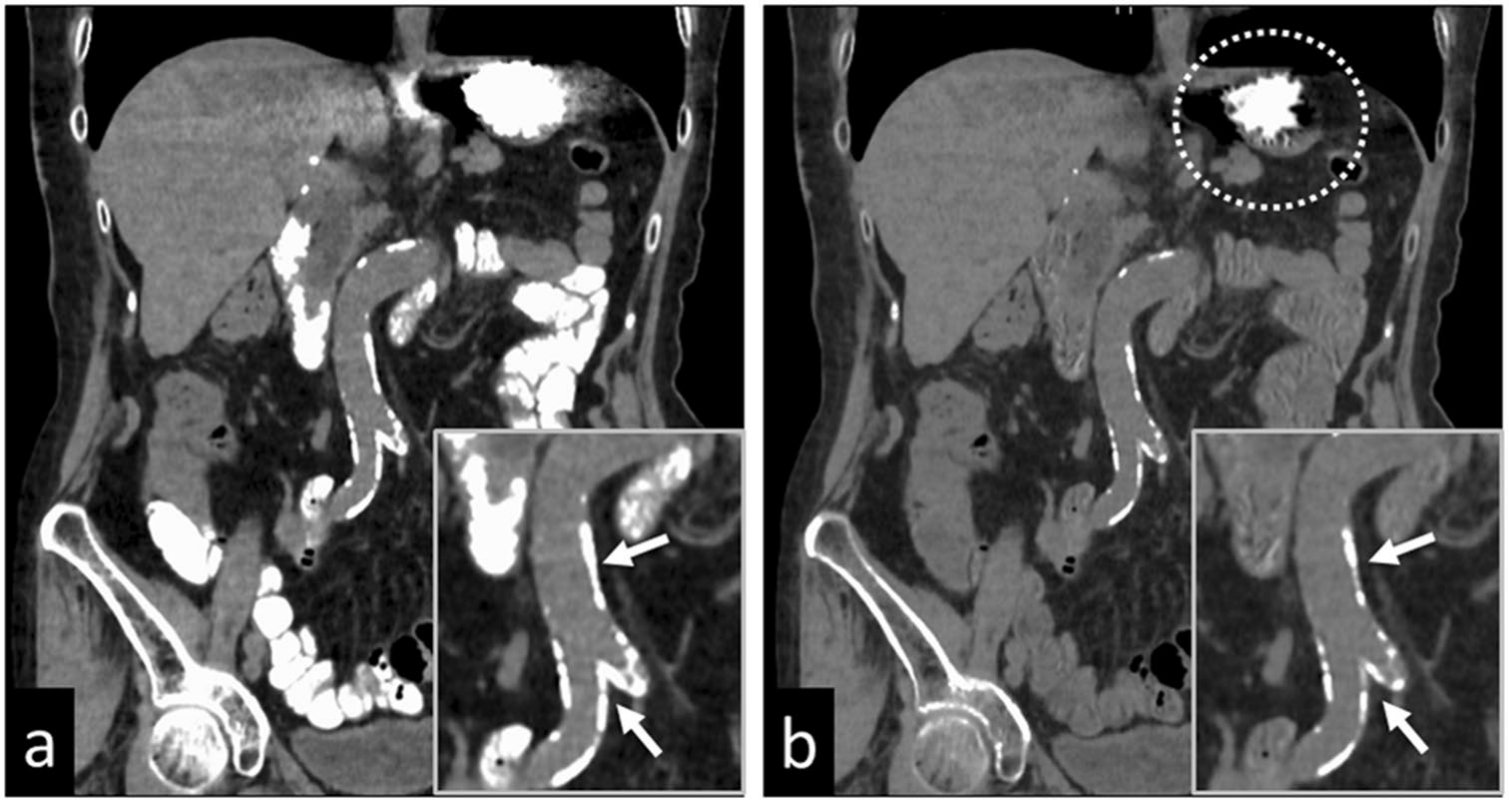
A 77-year-old woman who received gastrografin orally. The virtual monochromatic image at 70 keV (**a**) and the virtual non-contrast enhanced image (**b**) were obtained after unenhanced dual-energy CT. On the virtual non-contrast enhanced image (**b**), iodine in the small intestine is well removed. Iodine removal from the stomach is incomplete (dotted circle), suggesting that the iodine concentration was very high. During the virtual non-contrast reconstruction process, the volume of the calcification on the aortic wall was reduced (arrows)

However, the image quality of VNC images is decreased by a rough texture and poor spatial resolution. When the iodine concentration is very high, incomplete iodine removal is commonly observed [31]. In addition, tiny- and not highly attenuated calcific areas may be lost during the VNC reconstruction process [32] (Fig. 17).

### Edema images

Edema images generated from dual-energy CT scans are useful for the detection of early bone fractures [33–35] and acute ischemic stroke [36, 37].

**Fig. 18:**
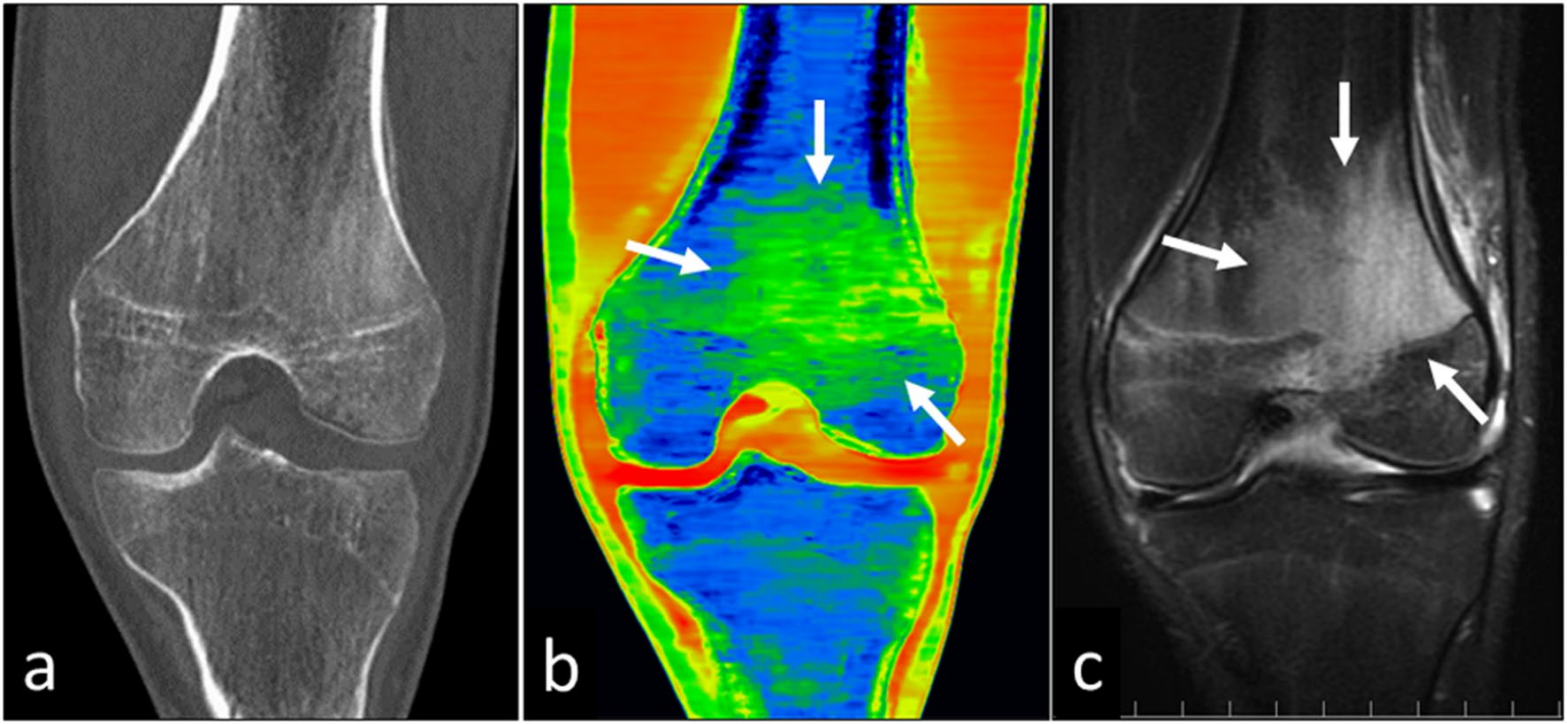
A 13-year-old man with a distal femur fracture. It is difficult to detect bone marrow edema on the virtual monochromatic 70 keV image (**a**). On the edema image (**b**), bone marrow edema (arrow) is visualized in the same area as on the short TI inversion recovery image (**c**)

Under the presumption that the human body contains water and calcium, in patients with early bone fractures, edema images can help to identify bone marrow edema (BME) (Fig. 18). By reducing the calcium signal from bone, water density images reflective of BME can be created. Lesions on BME images clearly reflect the water content in the bone marrow; these images have a high correlation with fat-suppressed T2-weighted images [33, 35]. The diagnosis of early bone fractures on BME images requires less time than does magnetic resonance imaging during which patients must be still for a prolonged time.

For the diagnosis and management of acute ischemic stroke, the detection of edema in the gray matter is essential; edema maps generated from dual-energy CT scans were reported to be useful [36, 37]. “X-map”, an application to identify acute ischemic lesions on non-contrast dual-energy CT scans [37], creates a virtual gray-matter- and water-content map using three-material decomposition. Lesions on the X-map clearly reflect the water content of cerebral edema induced by acute ischemic stroke. There is a good correlation between findings on X-maps and on diffusion-weighted images [37]. This method would be useful for the early development of treatment strategies in patients with acute ischemic stroke.

### Liver fat volume fraction and liver fibrosis estimation

**Fig. 19:**
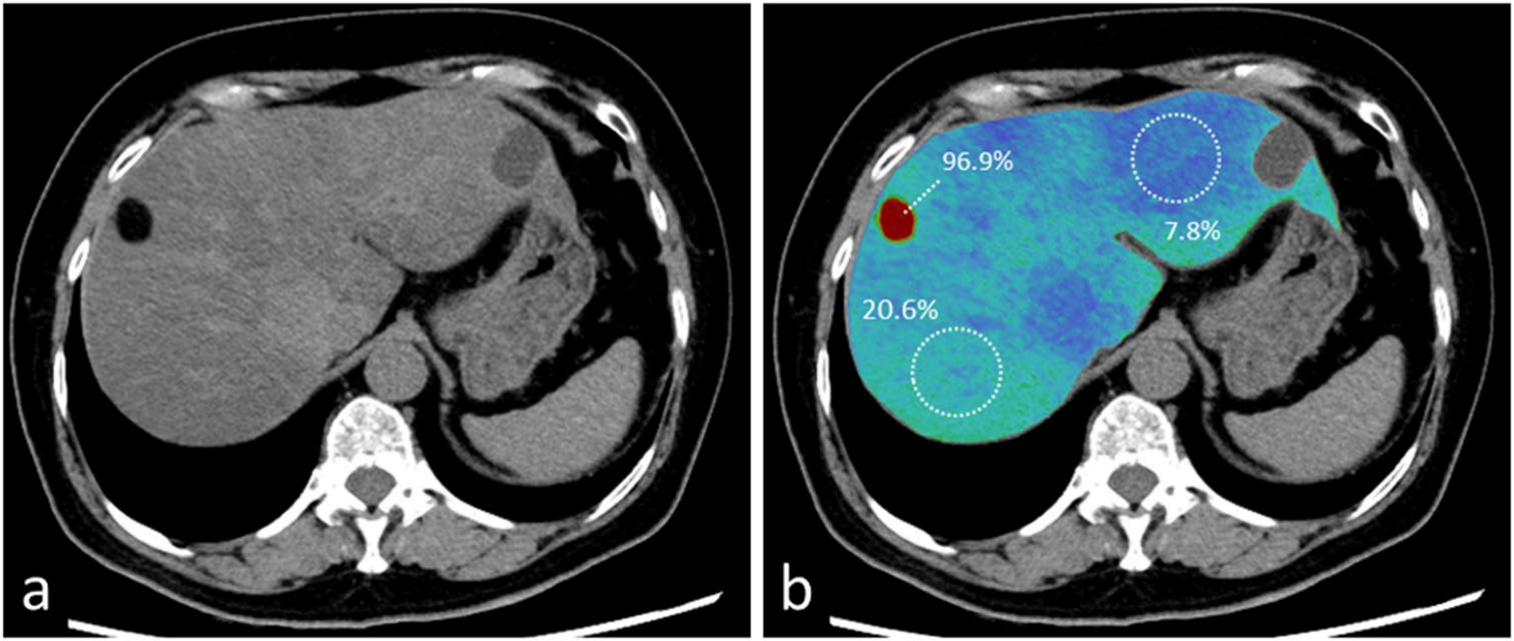
A 64-year-old woman with tuberous sclerosis, and focal hepatic steatosis and angiomyolipoma in the right lobe. A virtual monochromatic image obtained at 70 keV (**a**) and a color image of the fat map (**b**) are shown. The liver fat volume was 96.9% in the angiomyolipoma, 20.6% in the right-, and 7.8% in the left lobe of the liver

Multi-material decomposition algorithms facilitate the acquisition of the liver fat volume fraction (FVF) on dual-energy CT scans [38, 39]. It is presented as a volume % on fat maps and calculated directly from non-contrast dual-energy CT scans (Fig. 19). Fat maps from contrast-enhanced CT images are obtained after the creation of VNC images. According to Hyodo et al. [38], the liver FVF on non-enhanced- and dynamic contrast-enhanced dual-energy CT images is comparable to the FVF determined by using MR spectroscopy. This method can be expected to yield accurate and reproducible findings for the diagnosis of hepatic steatoses such as non-alcoholic- and alcoholic fatty liver disease. Validation of this method is required before its routine use in the clinical setting [40, 41].

Estimating the degree of liver fibrosis has been attempted using dual-energy CT data [42, 43]. Extracellular volume fraction calculated from iodine density images is reported to be useful in estimating the degree of liver fibrosis [42]. Also, CT texture analyses, such as gray-level intensity, skewness, kurtosis, and entropy at different energy levels are useful for the diagnosis of clinically significant hepatic fibrosis [43]. These parameters could be a promising biomarker of liver fibrosis; however, further research is needed for use in clinical examinations.

## Effective atomic number and electron density analysis

Effective atomic number- and electron-density analyses are based on raw data-based dual-energy analysis. The effective atomic number (effective *Z*) represents the average atomic number of a compound or mixture of materials. The electron density, on the other hand, represents the probability of an electron being present at a specific location. Highly accurate electron densities and effective atomic numbers have been calculated by raw data-based dual-energy analysis [13, 44], but their clinical applicability requires further investigations.

Because it is a promising method for obtaining electron density maps, dual-energy CT-based electron density imaging has attracted the interest of radiation oncologists. During the planning of radiotherapy, electron density maps are generated from single-energy CT scans to determine the dose distribution in the target tissues [45]. However, the CT number and the electron density of tissues are not accurately correlated because the CT number depends on not only the electron density but also the effective atomic number. Electron-density maps obtained from dual-energy CT scans were reported to be more accurate than the maps obtained with conventional radiotherapy planning methods [46, 47].

## Conclusions

This review presented the basics of dual-energy CT scanning and its usefulness in daily clinical practice. This technique makes it possible to identify the characteristics of materials that cannot be evaluated on conventional single-energy CT images. We think that familiarity with a wide variety of dual-energy CT applications and with their limitations facilitates the accurate interpretation of CT findings and helps to improve patient care in routine clinical practice.
